# Relationship between anthropometric and body composition parameters and anti-SARS-CoV-2 specific IgG titers in females vaccinated against COVID-19 according to the heterologous vaccination course: A cohort study

**DOI:** 10.1371/journal.pone.0287128

**Published:** 2023-06-13

**Authors:** Marlena Golec, Joanna Zembala-John, Martyna Fronczek, Adam Konka, Aneta Bochenek, Karolina Wystyrk, Hanna Botor, Marzena Zalewska, Martyna Chrapiec, Sławomir Kasperczyk, Zenon Brzoza, Rafał J. Bułdak

**Affiliations:** 1 Silesian Park of Medical Technology Kardio-Med Silesia, Zabrze, Poland; 2 Department of Medicine and Environmental Epidemiology, Faculty of Medical Sciences in Zabrze, Medical University of Silesia in Katowice, Zabrze, Poland; 3 Silesian Center for Heart Diseases, Zabrze, Poland; 4 Department of Pharmacology, Faculty of Medical Sciences in Zabrze, Medical University of Silesia in Katowice, Zabrze, Poland; 5 Acellmed Ltd., Zabrze, Poland; 6 Department of Basic Medical Sciences, Faculty of Public Health in Bytom, Medical University of Silesia in Katowice, Bytom, Poland; 7 Department of Biochemistry, Faculty of Medical Sciences in Zabrze, Medical University of Silesia in Katowice, Zabrze, Poland; 8 Department of Internal Diseases, Allergology, Endocrinology and Gastroenterology, Institute of Medical Sciences, University of Opole, Opole, Poland; 9 Department of Clinical Biochemistry and Laboratory Diagnostics, Institute of Medical Sciences, University of Opole, Opole, Poland; Waseda University: Waseda Daigaku, JAPAN

## Abstract

**Introduction:**

The aim of this cohort study was to evaluate the relationship between anthropometric and body composition parameters and anti-SARS-CoV-2 IgG titers in a group of females who were vaccinated against COVID-19 with two doses of ChAdOx1 vaccine and then boosted with the BNT162b2 vaccine.

**Materials and methods:**

The study group consisted of 63 women. Basic demographic and clinical data were collected. To assess the anti-SARS-CoV-2 immunoglobulin G titers following the vaccination, five blood draws were performed: 1) before the first dose, 2) before the second dose, 3) 14–21 days after the primary vaccination, 4) before the booster, and 5) 21 days after the booster. Blood samples were analyzed using a two-step enzymatic chemiluminescent assay. Body mass index and body composition were evaluated using bioelectrical impedance analysis. To select the most distinguishing parameters and correlations between anthropometric and body composition parameters and anti-SARS-CoV-2 IgG titers, factor analysis using the Principal Component Analysis was conducted.

**Results:**

Sixty-three females (mean age: 46.52 years) who met the inclusion criteria were enrolled. 40 of them (63.50%) participated in the post-booster follow-up. After receiving two doses of the ChAdOx1 vaccine, the study group’s anti-SARS-CoV-2 IgG titers were 67.19 ± 77.44 AU/mL (mean ± SD), whereas after receiving a heterologous mRNA booster, the level of anti-SARS-CoV-2 IgG titers was about three-times higher and amounted to 212.64 ± 146.40 AU/mL (mean ± SD). Our data shows that seropositivity, obesity, non-fat-related, and fat-related body composition parameters all had a significant effect on the level of IgG titer after a two-dose vaccination of ChAdOx1. However, only non-fat-related and fat-related body composition parameters had a significant effect on the IgG titer after booster vaccination.

**Conclusion:**

COVID-19 infection before the first dose of vaccination is not related to IgG titer after booster administration. Body composition has a significant effect on the production of anti-SARS-CoV-2 IgG after booster vaccination in females.

## Introduction

Vaccination has been considered the most impactful strategy for managing and controlling the coronavirus disease (COVID-19) pandemic caused by Severe Acute Coronavirus Syndrome 2 (SARS-CoV-2) [1–3]. Currently, several dosen vaccinees of different types has been approved and available globally [4–6]. Among the most frequently used products in Europe are the messenger ribonucleic acid (mRNA) vaccine BNT162b2 (hereinafter referred to as “BNT”; Pfizer/BioNTech) and the vector vaccine ChAdOx1 [recombinant] (AstraZeneca; hereinafter referred to as “ChAd”) [7–9]. Initially, depending on the product type, one or two doses of vaccine have been offered. However, reported findings on waning post-vaccinal immunity and decreased vaccines’ efficacy against emerging SARS-CoV-2 variants of concern have resulted in introduction of a booster dose to strengthen the protection against the coronavirus infection [10]. For individuals primed with two doses of ChAd, the BNT booster was recommended; a heterologous ChAd/ChAd/BNT vaccine scheme has been adopted in many countries, also in Poland [11–13].

Immune response to a given pathogen depends on many factors, i.a., the nature of an agent, inter-individual variability, and sex of a given subject. Multiple studies investigating immune system responses to vaccines against common communicable diseases have recognized and documented that phenomenon [14–17].

Females tend to respond faster to viral infections than males and develop stronger innate and adaptive responses (both humoral and cellular) to infection or vaccination than their male counterparts [16–18].

Emerging data suggest that also in the case of SARS-CoV-2, sex may significantly impact infection course and vaccine-induced immunity [19–21]. Knowledge on that topic, however, remains scarce.

Risk factors predisposing to severe COVID-19 outcomes have been more recognized, and they include male sex and, i.a., obesity-associated not as much with body mass index (BMI) solely as with excessive body fat accumulation, as it impairs the ability to produce antibodies against SARS-CoV-2 virus spike proteins [22–24].

It must be highlighted, however, that this indicator of obesity has certain limitations, as it does not consider the body type, does not distinguish between fat and lean mass, and does not provide information on the distribution of body fat [25]. Moreover, it does not consider existing sex differences [24, 26, 27]. For example, females usually have a lower BMI than males; however, their fat mass relative to their body type or BMI is considerably greater [25]. Also, females tend to have greater subcutaneous fat, while males–have visceral. Therefore, studies on the role of obesity in the context of the humoral immune response following SARS-CoV-2 vaccination should consider sex-related differences in adipose tissue and body fat distribution.

Since the beginning of the pandemic, many international organizations, including, i.a., the World Health Organization (WHO), the United Nations (UN), and European Commission (EC), called for the urgent need to adopt a sex- and gender-oriented perspective in research related to SARS-CoV-2 [28–30]. Still, many countries have not applied it fully in practice and have not collected sex-disaggregated data routinely [31].

In response to the global call for including a sex-oriented perspective in studies related to SARS-Cov-2, in our research, we decided to focus on women and their immune response to vaccination against SARS-CoV-2 on the timeline. In addition, since females have higher body fat levels than males, our study’s results could bring more light onto the role of adipose tissue in mechanisms of post-vaccinal immune response development.

Our study aimed to evaluate the influence of women’s age, metabolic age, selected anthropometric and body composition parameters (including body fat, lean mass, and body water), and their derived ratios (i.a., BMI and basal metabolic rate, i.e., BMR) on the magnitude of the anti-SARS-CoV-2 immunoglobulin G (IgG) titers after homologous primary vaccination with ChAd and heterologous boosting with the BNT vaccine in this group. In addition, we also wanted to verify whether being naïve before receiving the first vaccine dose could affect vaccine-induced humoral response in women.

## Materials and methods

### The study design and the study group

**Fig 1:**
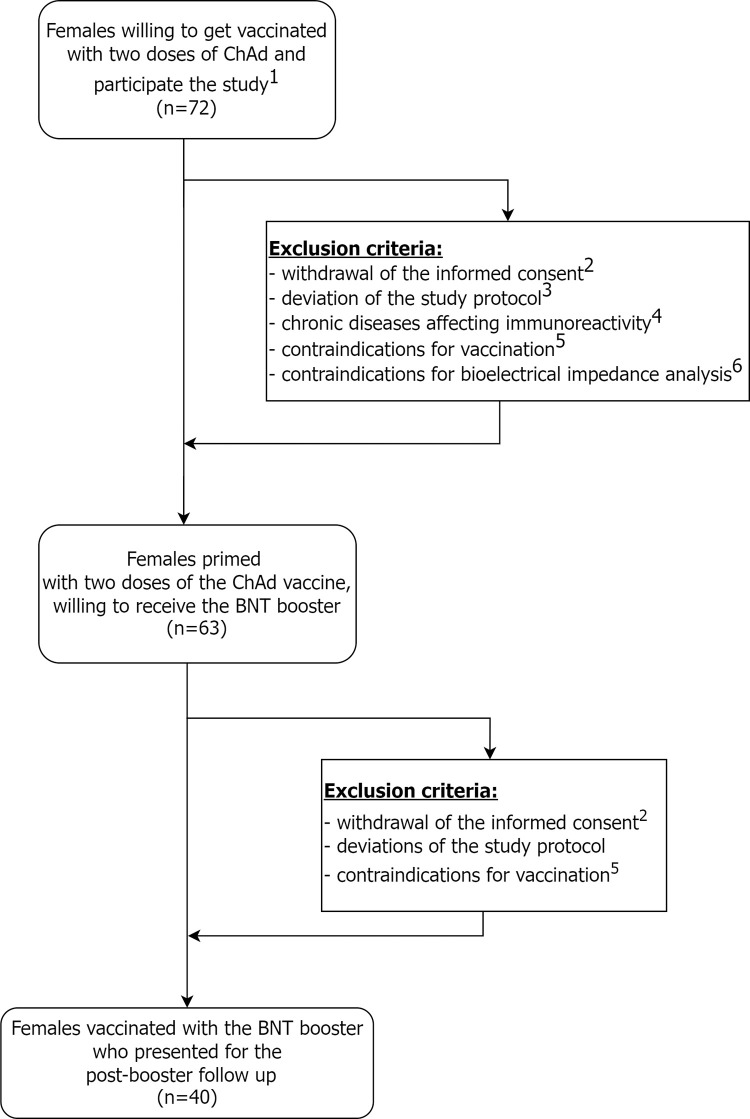
Study STROBE flow diagram of the study. 1—the presence of selected chronic diseases affecting immunoreactivity (i.e., autoimmune diseases, including autoimmune thyroiditis and chronic obstructive pulmonary disease); 2—deviations of the study protocol (i.a., not being able to participate in the blood collections according to the predicted blood collection protocol); 3—contraindications for vaccination (e.g., due to ongoing infection); 4—in case of anthropometric and body composition assessment—self-reported pregnancy, motor disabilities or implanted cardiac devices or other metal implants;5—withdrawal of the informed consent from the study (for personal or other reasons).

This cohort study was conducted in the Silesian Park of Medical Technology Kardio-Med Silesia (KMS) in Zabrze, Poland. Humoral response assessment was carried out in KMS Medical Laboratory, accredited by the Polish Center for Accreditation (Accreditation Certificate no. AB 1802). The study group consisted of females vaccinated against SARS-CoV-2 with two doses of the ChAd and boosted with the BNT vaccine (Fig 1). A heterologous vaccination schedule for ChAd-primed individuals has been recommended by the WHO Strategic Advisory Group of Experts on Immunization [9].

The inclusion criteria were: age ≥ 18 years old, female sex, willingness to undergo vaccination with two doses of the ChAd in a primary courseand to receive a booster dose of the BNT, approval to undergo whole blood draws to measure the immune response, and the consent for anthropometric and body composition measurements. All three vaccinations and all blood draw had to be performed at the study site.

Application of the above mentioned inclusion and exclusion criteria allowed the authors to create a relatively homologous study group and reduce a potential bias resulting from i.a., health status (e.g., presence of chronic diseases affecting immunoreactivity) or sex of the vaccinee, and sex-specific factors.

The first and the second dose of the ChAd vaccine was given with an interval of 8–12 weeks between the doses, and the booster with BNT vaccine was administered 6–7 months following the primary vaccination course. The vaccination program in the study group was carried out between March 2 and December 27, 2021: the first dose was administered between March 2–12, 2021, the second dose–between May 16–25, 2021, and the booster dose between November 26 and December 27, 2021. The interval between the second and the booster dose was dictated, besides WHO recommendations, obliging at that time and by the national vaccination schedule, indicating an interval of at least 6 months between those doses [32, 33].

**Fig 2:**
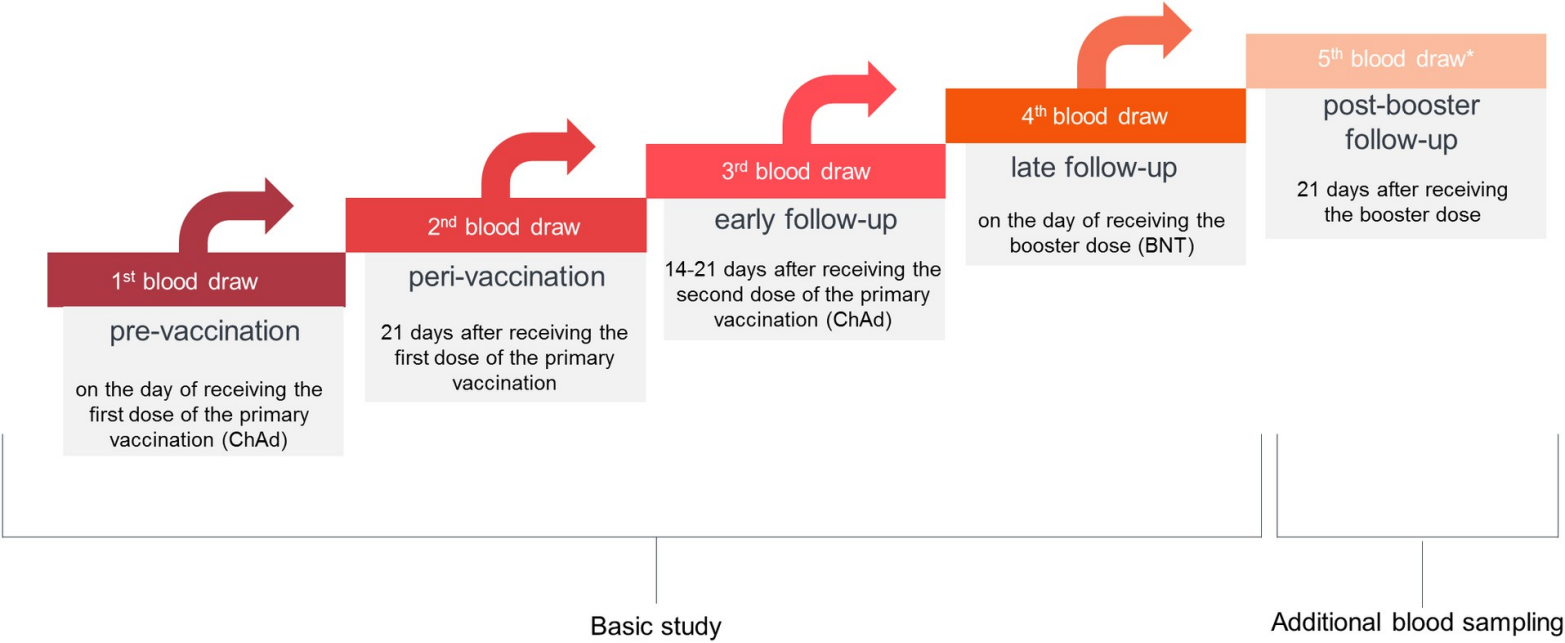
Schematic diagram of the sample collection. The first and the fourth blood draws were performed prior to the vaccine administration. *The 5th blood sampling was performed in a group of 40 out of 63 females. Abbreviations: ChAd—ChAdOx1 vaccine (AstraZeneca/Oxford), BNT–BNT162b2 (Pfizer/BioNTech).

According to the initial study protocol, the blood in the study group was collected four times: 1) before receiving the first vaccine dose, 2) 21 days after the first dose, 3) 14–21 days after the second vaccine dose, and 4) on the day of receiving the booster. During the study, we decided to extend the study protocol for additional (the fifth) blood draw to be performed 21 days after the booster. Out of 63 participants, 40 individuals presented for the fifth blood sampling. Due to the large sample size, we decided to perform an additional analysis of the post-booster data in this cohort (Fig 2).

The selection of the time points at which participants’ blood was collected was dictated by several reasons. First, many researchers test the immune response 21 days after the second vaccine dose [34, 35]. Moreover, many studies are based on a range of 14–35 days after vaccination or contracting COVID-19 [36, 37]. Finally, in our previous studies, assessing the humoral response to anti-SARS-CoV-2 vaccination among healthcare workers, the blood draws were performed between 14–21 days after the administration of vaccine doses [38, 39]. By adopting similar time intervals of blood sampling, it was possible to compare the results of the herein work with other studies.

The study was approved by the Insitutional Review Board of the Medical University of Silesia in Katowice (PCN/0022/KB1/50/II/20/21) and was conducted in accordance with the Helsinki Declatation.

All participants were informed about the concept and scope of the study and signed the informed consent form prior to enrollment. The written informed consent was obtained from all subjects prior to participating in the study.

### Participants’ demographic and clinical data

Participants’ demographic and clinical data, i.a., age, smoking, overweight, confirmed chronic diseases (including conditions associated with higher risk for severe COVID-19 outcomes, such as diabetes and hypertension), self-reported pregnancy, and eventual possession of metal implants were obtained through an author’s questionnaire. In addition, data on COVID-19 history (being naïve or convalescent before the study started), and the date of the SARS-CoV-2 test performed (only infection confirmed by the real-time reverse transcription-polymerase chain reaction (RT-PCR)) were collected.

### Anti-SARS-CoV-2 IgG measurement

To measure anti-SARS-CoV-2 IgG antibody titers, during each blood draw one aliquot of the whole blood was taken to the VACUETTE® TUBE 4 mL CAT Serum Clot Activator tube (Greiner Bio-One GmbH, Austria). Then, the samples were left at room temperature for 30 minutes to clot, and, next, they were centrifuged in Sorvall™ ST 16R Centrifuge (Thermo Fisher Scientific, USA) at 3500 rpm and temperature 2–8°C for 15 minutes to obtain sera. Each sample was analyzed using the ACCESS SARS-CoV-2 test in the Access 2 Immunoassay System Analyzer (Beckman Coulter Inc., USA). It is a two-step enzymatic chemiluminescent assay (CLIA) that detects IgG antibodies directed against the receptor-binding domain (RBD) of the S1 subunit of the spike protein (S) of the SARS-CoV-2 (SARS-CoV-2 S1-RBD). According to the manufacturer’s Instruction for Use (IFU), the analytical sensitivity of this assay regarding the limit of blank (LoB) and the limit of detection (LoD) is < 1.00 AU/ml and < 2.00 AU/ml, respectively. The lower limit of quantification (LoQ) for anti-SARS-CoV-2 IgG antibody concentration for this selected assay is 2.00 AU/mL and the upper limit is 8 000.00 AU/mL. The procedures and interpretation of the results were performed according to the manufacturer’s instructions. The samples were considered reactive when anti-SARS-CoV-2 IgG titers were ≥ 10.00 AU/mL, and non-reactive when they were < 10.00 AU/mL [40].

### Measurements of anthropometric and body composition parameters

Anthropometric and body composition parameters were measured using TANITA Body Composition Analyzer MC-780MA (TANITA Corporation, Japan). Participants’ BMI was calculated based on the measurement of body weight and height by using the formula: person’s weight (kg) divided by person’s height (m) squared. The results were interpreted according to the WHO classification, where BMI < 18.50 means underweight, 18.50–24.99—normal body weight, 25.00–29.99—overweight, and ≥ 30.00 –obesity [41]. Body composition was evaluated using TANITA bioimpedance analysis. Before the examination, the participant’s data (ID number, gender, date of birth, height, body type—standard vs. athletic) and the clothing weight were entered into the TANITA software—GMON program (GMON Pro 3.4.5, Medizin & Service GmbH, Chemnitz, Germany). A detailed description of the methodology applied in herein study was described in the authors’ previous work [38]. All measurements were conducted according to the manufacturer’s instructions and performed by the same study members.

### Statistical analysis

Data were presented as the mean (± standard deviation, SD) for variables with normal distribution and as median with a quartile range (Q1;Q3) for variables with non-normal distribution. To assess the normality of the distribution of quantitative variables, a graphical interpretation of the histogram and the QQ plot were used. Pearson’s linear correlation coefficient was applied to estimate the relation between parameters close to normal distribution. For anti-SARS-CoV-2 IgG titers assessments, a logarithmic transformation of data was performed to fit the normal distribution better. Due to the high amount of data and high correlations between the participant’s anthropometric and body composition parameters observed, factor analysis using the Principal Component Analysis (PCA) was conducted; then, based on the screen chart, distinguishing factors were selected. Subsequently, to maximize the percentage of explained variance, Varimax normalized factor rotation was used. Further analysis was performed using the values of the coefficients. To evaluate the correlation between the anti-SARS-CoV-2 IgG concentration and type of vaccine used (ChAd vs. BNT), age, and anthropometric and body composition parameters, a general linear model was applied. To assess the collinearity in the model, the variance inflation factor analysis (VIF) was conducted. The presence of autocorrelation was verified by Durbin-Watson’s test, while the analysis of the residuals was based on the verification of their mean and the conformity of the residual distribution with normal distribution. A homoscedasticity assessment was performed using a graphical method based on the residual plot. The analysis of influential observations was based on the Studentized residual values and Cook’s distance. In addition, to assess the influence of factors isolated in the PCA on changes in anti-SARS-CoV-2 IgG concentrations over time, the generalized estimation equation (GEE) model was used. This method was chosen due to existing autocorrelation of the anti-SARS-CoV-2 IgG measurements over time. Its results were presented as the coefficient value, standard error, Wald statistic value and p-value.

P values <0.05 were considered significant. All statistical analyses were conducted using Rstudio software (RStudio, PBC, Boston, MA, USA) [42].

## Results

Sixty-three women, who received two doses of ChAd vaccine during the primary vaccination course and were boosted with the BNT vaccine 6–7 months later were included in the study. The mean age in this group was 46.52 years (range: 22.00–64.00 years, SD ± 9.03). All subjects (100.00%) followed the initial study protocol, i.e. were vaccinated three times in KMS and participated in fourth blood draws. Only 40 of them (63.50%), however, showed up for the last, i.e., the fifth blood collection, performed after the booster administration. The study group’s basic demographic and clinical characteristics are presented below (Table 1).

**Table 1:** Basic demographic and clinical characteristics of the study group.

**Demographic and clinical characteristics, n = 63**		
**Parameters**	**n (%)**	
**age < 60 years**	57 (90.48)	
**age ≥ 60 years**	6 (9.52)	
**chronic diseases** ^**1**^	21 (33.33)	
**hypertension**	9 (14.29)	
**Allergy**	2 (3.17)	
**obesity (BMI ≥ 30)**	11 (17.46)	
**overweight (BMI ≥ 25.00)**	17 (26.98)	
**smoking**	6 (9.52)	
**Body composition characteristics, n = 63**		
**Parameters**	**Mean ± SD**	**Median (Q1; Q3)**
**TBW (kg)**	34.22 ± 5.34	33.20 (30.90; 36.80)
**ECW (%)**	14.95 ± 2.66	14.50 (13.20; 16.10)
**ICW (%)**	19.27 ± 2.84	18.90 (17.10; 20.80)
**BFP (%)**	28.96 ± 7.84	29.50 (23.50; 33.40)
**BFM (kg)**	21.19 ± 10.94	19.70 (13.80; 26.50)
**FFM (kg)**	48.11 ± 7.50	46.70 (43.40; 51.80)
**PMM (kg)**	45.67 ± 7.13	44.30 (41.20; 49.20)
**BBM (kg)**	2.44 ± 0.38	2.40 (2.20; 2.70)
**impedance (Ohm)**	638.30 ± 74.09	655.00 (596.00; 696.00)
**BMR (kJ)**	6007.46 ± 995.98	5749.00 (5443.00; 6557.00)
**VAT (level)**	5.65 ± 2.98	5.00 (3.00; 7.00)
**Anthropometric characteristics, n = 63**		
**Parameters**	**Mean ± SD**	**Median (Q1; Q3)**
**WC (cm)**	86.11 ± 15.96	83.00 (74.00; 96.00)
**HC (cm)**	104.54 ± 12.82	100.00 (97.00; 109.00)
**weight (kg)**	69.78 ± 17.33	66.30 (57.70; 77.90)
**height (cm)**	164.73 ± 6.64	164.00 (160.00; 170.00)
**BMI (kg/m** ^**2**^ **)**	25.64 ± 5.78	24.17 (21.07; 28.55)
**Pre-, peri- and post-primary vaccination anti-SARS-CoV-2 IgG titer, n = 63**		
**Parameters**	**Mean ± SD (AU/mL)**	**Median (Q1; Q3) (AU/mL)**
**before the first dose** ^**2**^	10.22 ± 24.86	0.52 (0.22; 6.38)
**after the first dose** ^**3**^	91.55 ± 113.72	50.61 (13.26; 141.25)
**early follow-up** ^**4**^	67.19 ± 77.44	40.57 (24.46; 79.55)
**late follow-up** ^**5**^ **before the booster dose**	61.14 ± 160.65	12.34 (5.17; 34.86)
**Post-booster anti-SARS-CoV-2 IgG titer, n = 40**		
**Parameters**	**Mean ± SD (AU/mL)**	**Median (Q1; Q3) (AU/mL)**
**post-booster follow-up** ^**6**^	212.64 ± 146.40	177.98 (120.25; 264.45)

^1^chronic diseases—chronic conditions other than hypertension, obesity, and allergy, and excluding diseases affecting immunoreactivity, described in the Exclusion criteria in Material and methods; ^2^before the first dose–anti-SARS-CoV-2 IgG titer measurement on a day of administration ot the first dose (ChAd); ^3^after the first dose—anti-SARS-CoV-2 IgG titer measurement 21 days after the first dose (ChAd); ^4^early follow-up—anti-SARS-CoV-2 IgG titer measurement 14–21 days after completing primary vaccination course (ChAd/ChAd); ^5^late follow-up—anti-SARS-CoV-2 IgG titer measurement on a day of administration of the booster dose (BNT); ^6^post-booster follow-up—anti-SARS-CoV-2 IgG titer measurement 21 days after receiving the booster vaccine.

Abbreviations: TBW–total body water; ECW—extracellular water; ICW—intracellular water; BFP—body fat percentage; BFM—body fat mass; FFM—fat-free mass; PMM—predicted muscle mass; BMM—body muscle mass; BMR—basal metabolic rate; VAT—visceral adipose tissue; WC—waist circumference; HC—hip circumference; BMI—body mass index.

**Fig 3:**
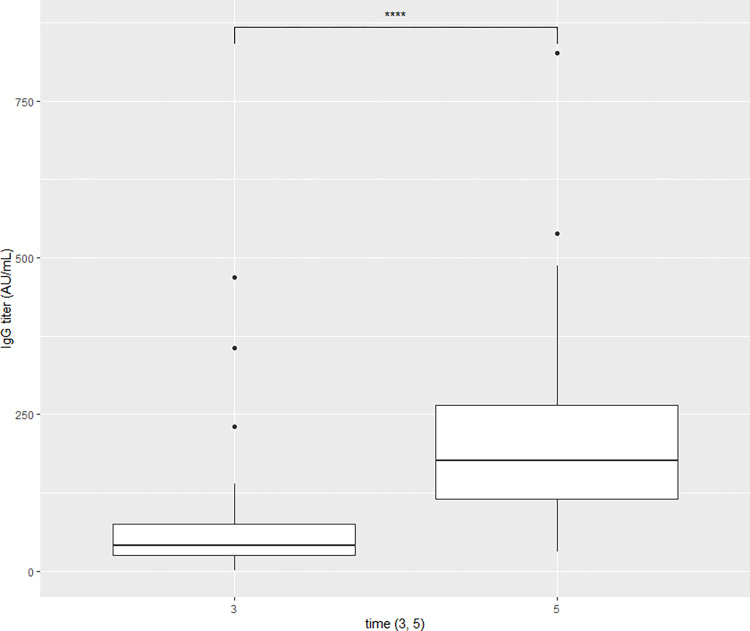
Comparison between anti-SARS-CoV-2 IgG titers at the 3^rd^ time point (3^rd^ blood draw–early follow-up, 14–21 days after receiving the second ChAd dose) and at 5^th^ time-point (5^th^ blood draw–post-booster follow-up, 21 days after receiving the BNT booster).

A comparison of the participants’ humoral response on the timeline revealed a significant rise in the concentration of anti-SARS-CoV-2 IgG antibodies at the 3^rd^ time point (14–21 days after the second dose of ChAd) and at the 5th time point (21 days after the BNT booster). Therefore, in our analyses, we decided to concentrate on those two peak level measurements. After the primary vaccination with 2-doses of homologous vector ChAd vaccine, anti-SARS-CoV-2 IgG titers in the study group was 67.19 ± 77.44 AU/mL (mean ± SD), while after receiving a heterologous mRNA booster, the level of anti-SARS-CoV-2 IgG titers was about 3-times higher and amounted to 212.64 ± 146.40 AU/mL (mean ± SD) (Fig 3).

**Fig 4:**
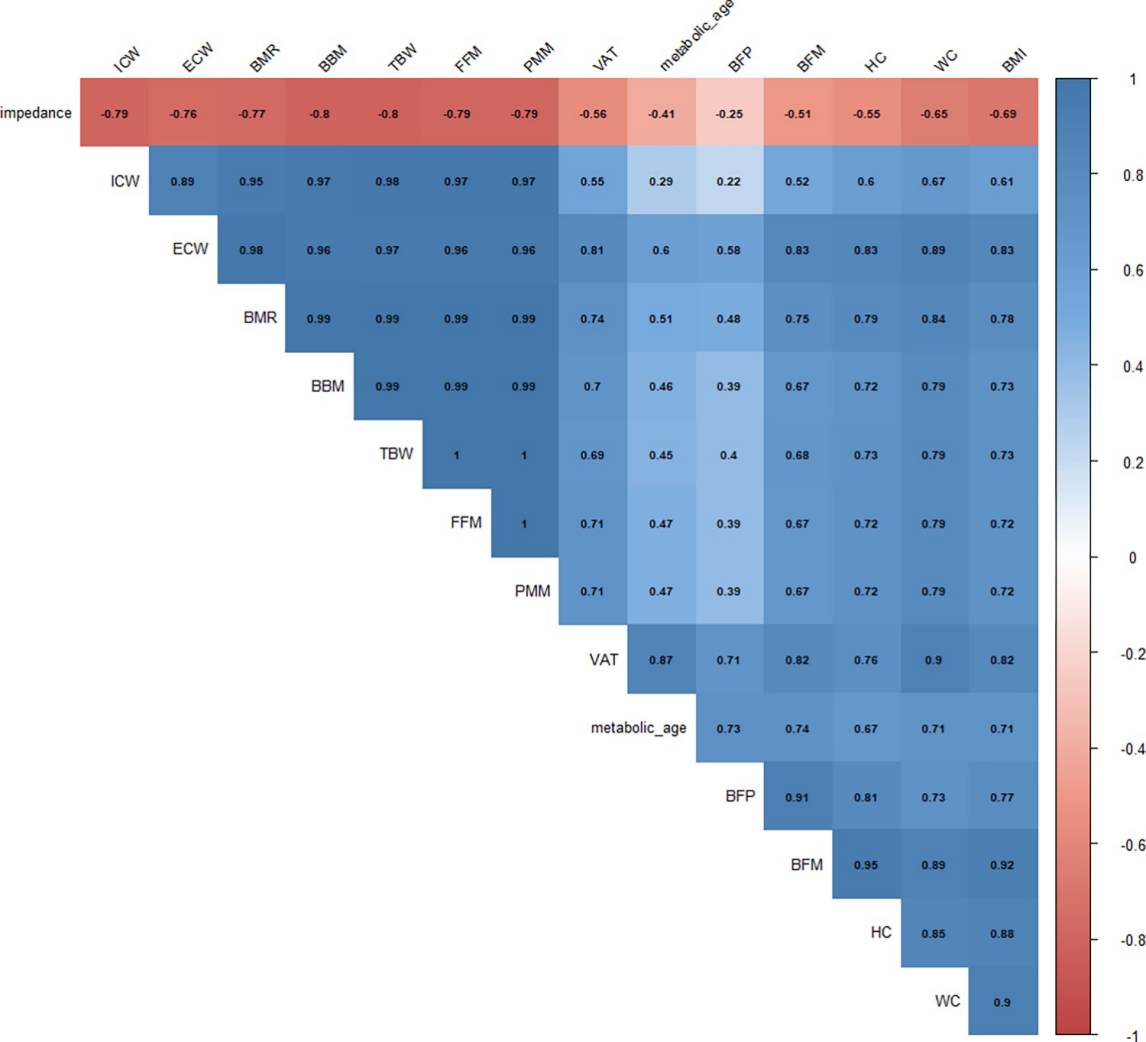
Heatmap of the correlation matrix generated by the Pearson r correlation coefficient values between selected anthropometric parameters. Abbreviations: ECW—extracellular water, ICW—intracellular water; BFP—body fat percentage; BFM—body fat mass; FFM—fat-free mass; TBW–total body water, PMM—predicted muscle mass; BBM—body bone mass; BMR—basal metabolic rate; VAT—visceral adipose tissue; WC—waist circumference; HC—hip circumference; BMI—body mass index.

During the first assessment of anti-SARS-CoV-2 IgG antibodies (before the administration of the first vaccine dose), non-normal distribution was reported, therefore for further analyses, a non-parametric test was applied. Not normal distribution was also observed in the third and fifth measurement points, however, after applying the logarithm, normality was obtained and the Pearson’s correlation coefficient was used (Fig 4).

**Fig 5:**
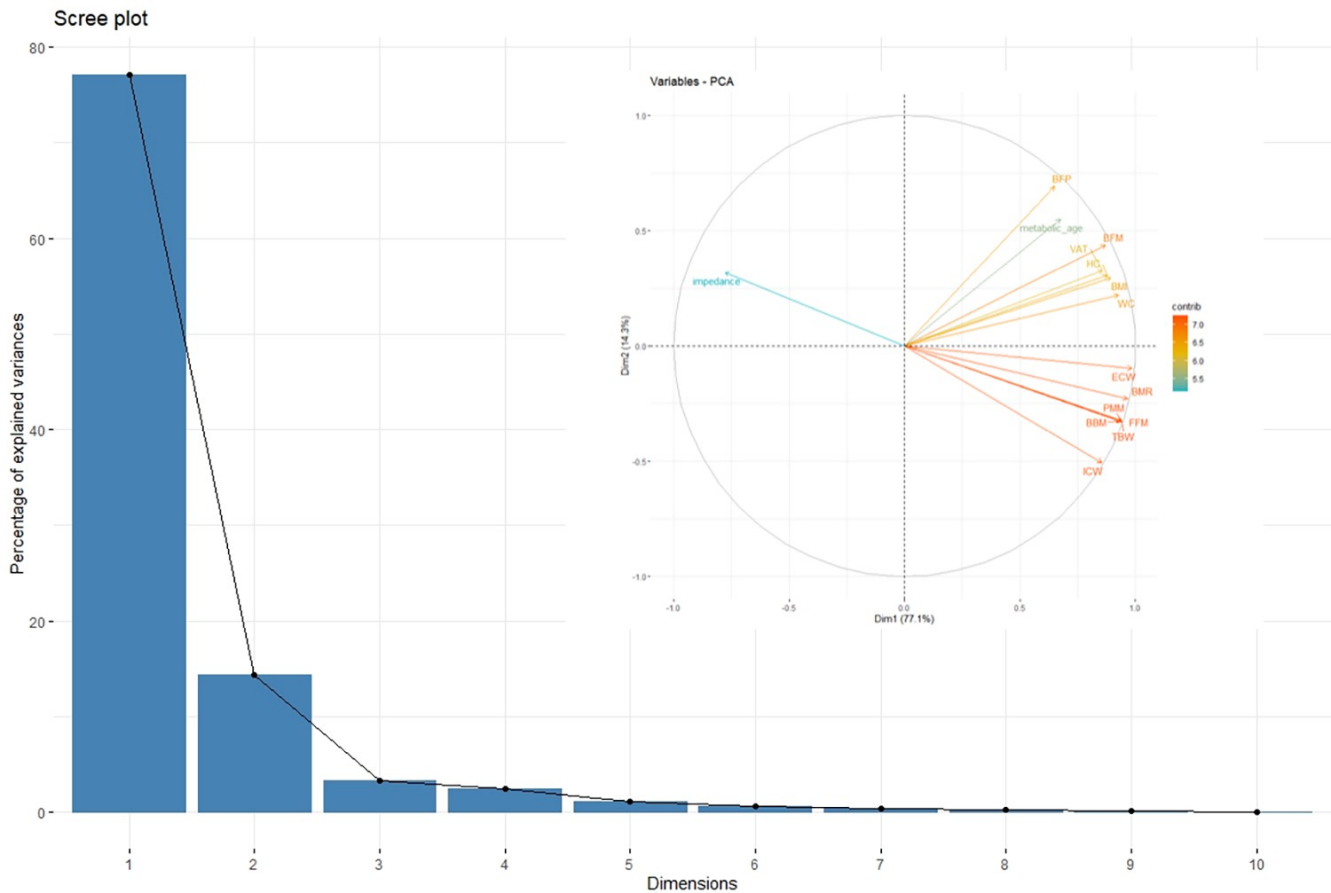
Principal component analysis. A. A scree plot and percentage of total variance accounted for by each factor. B. Factor loading plot of the principal component analysis (PCA) for selected anthropometric and body composition parameters. Abbreviations: TBW–total body water; ECW—extracellular water; ICW—intracellular water; BFP—body fat percentage; BFM—body fat mass; FFM—fat-free mass; PMM—predicted muscle mass; BMM—body muscle mass; BMR—basal metabolic rate; VAT—visceral adipose tissue; WC—waist circumference; HC—hip circumference; BMI—body mass index.

After primary 2-dose ChAd vaccination followed by the BNT booster, for collected anthropometric and body composition data a PCA was conducted. It is a method applied in the analysis of multiple variables that allows to reduce dimensionality of datasets, but at the same time–to retain information necessary to understand hidden structures and patterns [43]. In our study, all collected anthropometric and body composition parameters were closely correlated with each other. The results of PCA, indicating the proportion of variance against its eigenvalue rank (Scree plot) and factor loadings for selected anthropometric and body composition parameters (Factor loadings plot) are presented below (Fig 5).

High correlations between certain body composition parameters, i.e., body fat, body water (TBW, ICW, ECW) and waist circumference (WC), hip circumference (HC) and fat-free mass (FFM) were observed, which allowed us to perform a factor analysis that explained 92% of the variance.

We divided the analyzed variables into two groups, i.e., non-fat-related–called “non-fat factors” (NFF), and fat-related, called “fat factors” (FF). NFF consisted of parameters related to the supply of water, i.e., TBW, ECW, and ICW, and non-fat body substances, i.e., body bone mass (BBM), predicted muscle mass (PMM), and fat-free mass (FFM). FF, in turn, indicated fat-related parameters, i.e., body fat percentage (BFP), body fat mass (BFM), visceral adipose tissue (VAT), WC and HC as well as metabolic age, and BMI. In the case of the latter, a weaker relationship was observed; stronger correlations were noted for pure fat parameters, i.e. BFP, BFM, and VAT. It is worth highlighting that FF was positively correlated with obtained results: an increase in FF was associated with an increase in body fat (Table 2).

**Table 2:** Factor loadings were obtained from the PCA analysis.

Variables	Non-fat factor (NFF)	Fat factor (FF)
eigenvalue	12.16	1.72
% variance	81.04	11.44
cumulative variance	81.04	92.48
TBW (kg)	**0.92**	0.39
ECW (%)	**0.80**	0.58
ICW (%)	**0.97**	0.20
BFP (%)	0.26	**0.90**
BFM (kg)	0.54	**0.82**
FFM (kg)	**0.91**	0.40
PMM (kg)	**0.91**	0.40
BBM (kg)	**0.91**	0.38
impedance (Ohm)	**-0.81**	-0.25
BMR (kJ)	**0.88**	0.47
VAT (level)	0.37	**0.88**
metabolic age (years)	0.15	**0.89**
WC (cm)	0.56	**0.77**
HC (cm)	0.61	**0.71**
BMI (kg/m2)	0.58	**0.75**

Abbreviations: PCA—Principal Component Analysis; TBW—total body water; ECW—extracellular water; ICW—intracellular water; BFP—body fat percentage; BFM—body fat mass; FFM—fat-free mass; PMM—predicted muscle mass; BBM—body bone mass; BMR—basal metabolic rate; VAT—visceral adipose tissue; WC—waist circumference; HC—hip circumference; BMI—body mass index.

### The influence of selected variables on the anti-SARS-CoV-2 IgG antibody concentrations 14–21 days after two doses of ChAd vaccine–results of post-primary vaccination early follow-up (n = 63)

Then, the univariate analysis was applied for all model components, investigating each variable independently. A seropositivity before the primary vaccination (B = 0.21, p = 0.001), obesity (B = 0.15, p = 0.037), NFF (B = 0.11, p = 0.026), and FF (B = 0.13, p = 0.02) had a significant effect on the level of anti-SARS-CoV-2 IgG antibody measured 14–21 days after administration of the second ChAd dose (post-primary vaccination early follow-up). Smoking appeared to be insignificant–a borderline significance was reported (B = -0.17, p = 0.072) (Table 3).

**Table 3:** Univariate and multivariate linear regression analysis: Standardized Beta coefficients of independent variables assessed in the study group after receiving a 2nd dose of the ChAd vaccine (n = 63) and in the cohort participating in the post-booster follow-up (n = 40).

**Parameters**	**Early follow-up– 14–21 days after receiving 2nd dose of ChAdOx1 vaccine, n = 63**					
	**Univariate analysis**			Multivariate analysis* (adjusted R^2^ = 0.23)		
	**B**	**SE**	**p**	**B**	**SE**	**p**
**age ≥ 60**	-0.01	0.10	0.953	-0.02	0.11	0.869
**seropositivity before the study started** ^1^	**0.21**	**0.06**	**0.001**	**0.17**	**0.06**	**0.008**
**smoking**	-0.17	0.09	0.072	0.12	0.09	0.167
**allergy**	0.06	0.13	0.654	0.00	0.12	0.967
**obesity**	**0.15**	**0.07**	**0.037**	0.00	0.10	0.995
**NFF**	**0.11**	**0.05**	**0.026**	0.11	0.05	0.057
**FF**	**0.13**	**0.05**	**0.020**	**0.14**	**0.06**	**0.036**
**Parameters**	**Post-booster follow-up—21 days after receiving the BNT162b2 booster, n = 40**					
	**Univariate analysis**			Multivariate analysis** (adjusted R^2^ = 0.19)		
	**B**	**SE**	**p**	**B**	**SE**	**p**
**age ≥ 60**	0.09	0.08	0.258	0.10	0.09	0.272
**seropositivity before the study started** ^**1**^	**0.13**	**0.05**	**0.027**	0.06	0.06	0.333
**smoking**	-0.12	0.07	0.079	0.07	0.06	0.250
**allergy**	-0.01	0.11	0.962	-0.02	0.10	0.854
**obesity**	**0.15**	**0.06**	**0.022**	0.01	0.10	0.886
**NFF**	**0.09**	**0.04**	**0.022**	0.09	0.05	0.094
**FF**	0,08	0.05	0.086	0.06	0.06	0.319

^1^seropositivity before the study started–anti-SARS-CoV-2 IgG titers ≥ 10.00 AU/mL before the first vaccine dose

*Durbin-Watson parameter is equal 1.87

**Durbin-Watson parameter is equal 2.04.

Abbreviations. R–linear regression coefficient, B—logistic regression coefficient, SE–standard error, p–statistical significance, NFF—non-fat factor, FF—fat factor.

In turn, in the multivariate analysis, two strategies were used to check the influence of the tested variables on the level of anti-SARS-CoV-2 IgG titers. First, we analyzed simultaneously all the factors. Seropositivity before the first vaccine dose and FF were statistically significant; this model, however, was weak (linear regression coefficient R^2^ = 0.23). The remaining variables tested in this model turned out to be insignificant. The results of the univariate and multivariate analysis are presented below (Table 3).

Next, a multiple linear regression using the backward elimination method was performed. A model consisting of three variables: NFF, FF, and being convalescent before the first dose was constructed. Results of performed analysis demonstrated that those three factors had the strongest effect on the level of anti-SARS-CoV-2 IgG antibody concentration 14–21 days after the primary vaccination course. The created model explained 26.00% of the adjustment (Table 4).

**Table 4:** Multiple linear regression using the backward elimination method.

**Parameters**	**Early follow-up—14–21 days after receiving the second ChAd vaccine n = 63**			
	**B**	**SE**	**P**	**Adjusted R** ^**2**^
seropositivity before the study started*	0.19	0.06	0.002	0.26
**NFF**	0.11	0.05	0.022	
**FF**	0.11	0.05	0.020	
**Parameters**	**Post-booster follow-up—21 days after receiving the BNT booster vaccine** **n = 40**			
	**B**	**SE**	**P**	**Adjusted R** ^**2**^
**NFF**	0.10	0.04	0.006	0.20
**FF**	0.11	0.04	0.019	

*seropositivity before the study start—anti-SARS-CoV-2 IgG titers ≥ 10.00 AU/mL before the first vaccine dose. Abbreviations: R–linear regression coefficient, B—logistic regression coefficient, SE—standard error, p—statistical significance, NFF—body non-fat factor, FF- body fat factor.

### The influence of selected variables on the anti-SARS-CoV-2 IgG titer after two doses of ChAd vaccine followed by the BNT booster–results of post-booster follow-up (n = 40)

Similarly, also for the cohort participating in the post-booster follow-up, the univariate analysis was performed. It was applied for all model components, analyzing each variable independently. Seropositivity before the study started (B = 0.13, p = 0.027), obesity (B = 0.15, p = 0.022), and NFF (B = 0.09, p = 0.022) had a significant effect on the level of anti-SARS-CoV-2 IgG concentrations after the intake of two doses of ChAd vaccine followed by the BNT booster. In multivariate analysis, on the other hand, all analyzed variables were statistically insignificant, and R^2^ = 0.19 (Table 3).

The regression analysis using the stepwise model allowed us to create a model consisting of NFF and FF without considering seropositivity before the study started, R^2^ = 0.196. The analysis demonstrated that both NFF and FF had the strongest effect on the amount of anti-SARS-CoV-2 IgG antibody concentration after the booster. The constructed model explained 20.00% of the fit of only 2 variables (Table 4).

### The influence of factors isolated in the PCA on changes in anti-SARS-CoV-2 IgG titer after booster administration (n = 40)

**Fig 6:**
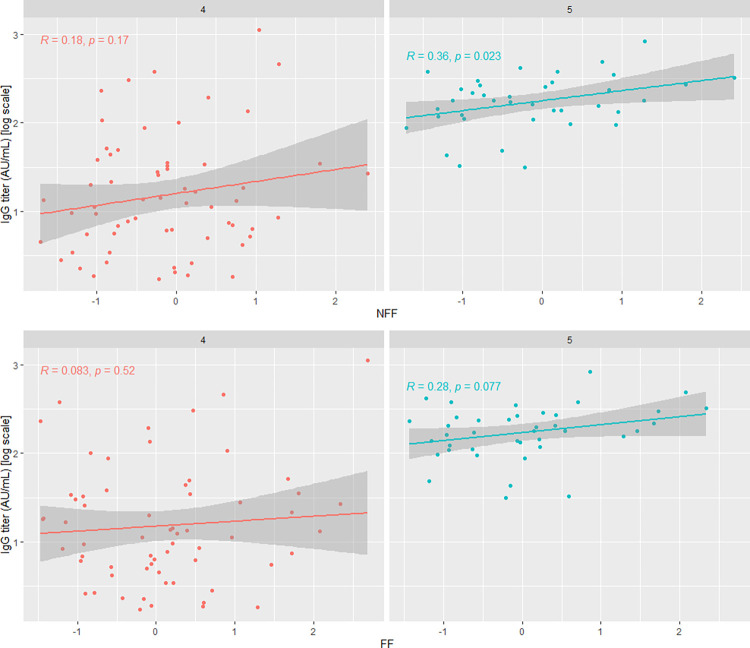
Correlation plots between anti-SARS-CoV-2 IgG antibody concentrations and tested factors (NFF and FF) before (4) and after (5) booster administration. Results of GEE model.

In the GEE model for changes in anti-SARS-CoV-2 IgG titers after the booster, a significant effect of NFF on the antibody concentration after the booster was observed (time). Patients with higher NFF values had, on average, higher anti-SARS-CoV-2 IgG antibody concentrations (Table 5, Fig 6).

**Table 5:** Impact of non-fat factors and fat factors on anti-SARS-CoV-2 IgG titers after the BNT booster. GEE model results.

**Variables**	**Estimate**	**SE**	**Wald**	**P**
	-3.02	0.44	46.67	< 0.001
**Booster administration**	1.05	0.09	131.41	< 0.001
**NFF**	0.12	0.05	5.37	0.021
**FF**	0.05	0.06	0.7	0.404

## Discussion

Since the beginning of the COVID-19 outbreak, numerous studies have been conducted to investigate the natural history of SARS-CoV-2 infection and the factors affecting its course and outcomes. With an increasing number of new cases and deaths, it has become clear that sex (understood as biological characteristics) plays an essential role in the epidemiology of COVID-19. According to global COVID-19 statistics, SARS-CoV-2 has affected a disproportionally higher number of males: although the morbidity rate was similar between sexes (slightly higher in men), the mortality rate was much higher among males [44].

With the above-mentioned facts in mind, international health authorities, including i.a. the United Nations, World Health Organization, and European Commission urged adopting a sex-oriented perspective in research related to COVID-19 [30, 45, 46]. Shared among the countries sex-disaggregated data at the initial stage of the COVID-19 pandemic has led to a better understanding of coronavirus disease incidence and mortality [28, 47]. Many countries, however, have not applied this recommendation fully in routine practice [46]. Therefore, despite a growing body of evidence on sex-related differences regarding SARS-CoV-2, knowledge on this topic remains limited.

Numerous studies conducted before the COVID-19 pandemic pointed out differences between the male and female immune system and their responses to infection or vaccination. According to the researchers, sex affects many components of the innate and adaptive immune system, including i.a. production of immunoglobulins and activation of T cells (higher in women) and cytokines, especially pro-inflammatory ones (higher in men) [48, 49].

Among the factors responsible for sex disparities mentioned above are, i.a., differences in the hormonal system (i.e., the protective effect of estrogens that affect immunocompetence) and genetics (sex chromosome genes—the expression of genes linked to the X chromosome [14, 48, 50–53].

Those observations were also confirmed in regard to COVID-19. It is known that SARS-CoV-2 enters the host cells by binding the receptor-binding domain of the viral Spike protein to the angiotensin-converting enzyme-2 receptor (ACE2) [54]. Estrogens suppress viral replication by upregulating the expression of ACE2 [55]. Androgens (testosterone and dihydrotestosterone), in turn, bind to androgen receptors and enhance the RNA polymerase activity, facilitating the entry of SARS-CoV-2 into the host’s cells [54]. This phenomenon is reflected in the higher susceptibility of males to infective agents and the development of the disease. In contrast, females are more resistant to infections and react faster and more robustly to the given pathogen [18, 56].

More severe COVID-19 course and higher mortality rate among men can also be partially explained by differences in overall health status and health behaviors between sexes: higher prevalence of chronic conditions (i.a. cardiovascular, metabolic, and respiratory diseases) and risk factors (smoking, excessive drinking), among men compared to women. It has been documented that those conditions are associated with a higher risk of severe COVID-19 outcomes, including death.

A mounting body of evidence indicates that the subject’s sex also determines their immune response to vaccination. For example, sex-driven differences have been observed earlier in immunization against i.a., influenza, herpes virus, and other common infectious diseases [18, 57–59]. In addition, it was found that females induce and maintain higher humoral and cellular responses to vaccination than their male counterparts [17].

The above-mentioned reports have sparked the debate on introducing sex-specific changes in vaccination policy, which continued during the COVID-19 pandemic [60, 61]. Many researchers have highlighted potential benefits that optimization of vaccination strategy, considering sex- and gender-specific differences could bring [24, 61]. Since the main emphasis in SARS-CoV-2 studies was placed on males as more vulnerable to COVID-19, we decided to concentrate on females—their response to vaccination against COVID-19 and factors affecting its magnitude on the timeline. Our research may constitute valuable input to this discussion.

Our study investigated different variables that could affect post-vaccinal humoral immunity, among others, sex-specific anthropometric and body composition parameters.

Obesity has been identified as one of the most significant risk factors for severe COVID-19 outcomes. In addition, some studies also suggest the negative impact of excessive body mass on vaccine efficacy [62–64]. The most common tool used to define adiposity is body mass index (BMI). However, as mentioned earlier, this metric’s reliability is limited, as it does not consider, i.a., body type and sex-specific body composition.

It has been well documented that body structure and composition differ significantly between sexes. Generally, females have higher amounts of adipose tissue than males (20.00–25.00% vs. 10.00–15.00%) and lower muscle mass than their male counterparts [65, 66]. Moreover, females have higher subcutaneous fat, while males have higher visceral fat [66, 67]. Another significant difference constitutes fat distribution: in women, adipose tissue is located mainly in the lower part of the body (buttocks, tights), while in men–in the upper, in abdominal region [68]. Fat cells’ location is crucial as it determines their function in health and disease [69].

Besides storing and releasing energy, adipose tissue also acts as a highly active endocrine organ that dynamically affects overall health conditions [70–72]. Moreover, it is strictly involved in coordinating multiple processes, including those related to the immune system functioning [73].

Adipose tissue, visceral in particular, has been shown to express ACE2 –a functional receptor used by SARS-CoV-2 to enter the host cells [74]. Moreover, it serves as a SARS-CoV-2 reservoir and its replication site [75]. Excessive adipose tissue may lead to higher viral overload and slower immune response to the pathogen, which could partially explain the more severe COVID-19 course and higher mortality due to COVID-19 in obese individuals [76]. Since males usually have higher visceral fat, they express more ACE2; hence, we can assume that they provide potentially more favorable conditions for SARS-CoV-2 invasion.

Expansion of adipose tissue leads to its dysfunctions and is linked, among others, to its proinflammatory activity [62, 69, 77]. As body fat (visceral in particular) produces cytokines (including proinflammatory proteins, such as i.a., interferon-γ, TNF-α, IL-1, and IL-12), its excessive amount results in their excessive secretion and increase in the blood, which may lead to “cytokine storm” [62, 78, 79]. Skeletal muscle also plays a pivotal role in the immune response to infection, as it produces anti-inflammatory and immunoprotective substances called myokines that help to alleviate exacerbated inflammation in COVID-19 [80, 81].

Obesity may also affect vaccine-induced immune response. Data on that topic are, however, inconclusive. Numerous studies report lower vaccine efficacy among obese individuals, however, some papers indicate a lack of such association [82–84]. Watanabe et al., for example, in their assessment of IgG antibodies after primary vaccination with the BNT vaccines, did not find a correlation between BMI and anti-SARS-CoV-2 IgG titers [85]. Pellini et al., in turn, in their research on the humoral response measured seven days after the primary vaccination, reported higher anti-SARS-CoV-2 IgG concentrations in individuals with normal body mass compared to obese ones. In multivariate linear regression analysis, however, BMI in those groups had no significant impact on the level of post-vaccinal anti-SARS-CoV-2 IgGs [86]. It is worth highlighting that in the above-cited studies obesity was defined by BMI and that they were conducted on the general population. As mentioned earlier, BMI has some limitations; among others, it does not consider sex-specific fat distribution, which may bias the results. It also is worth stressing that the mean percentage of body fat in females with normal body mass (BMI 18.00–25.00) is comparable to the mean percentage of body fat in males who are obese (BMI >30.00) [87].

Our study group consisted of females only; therefore, we could exclude fluctuations resulting from sex-related differences in body composition (fat and lean mass). Moreover, our research investigated the impact of adiposity determined not only by BMI classification. We also analyzed the role of the body composition using bioelectrical impedance analysis (BIA). This method identifies, i.a., body fat level more precisely than simple BMI [88]. Therefore, it constitutes a more reliable tool for determining the impact of adiposity on immune response after vaccination against COVID-19.

The results of our study revealed that selected anthropometric and body composition parameters substantially affected vaccine-induced humoral response in females. We found that both non-fat factors (NFF) and fat factors (FF) of the body had a significant impact on the level of anti-SARS-CoV-2 IgG titers: increased fat mass and increased muscle and bone mass translated onto higher post-vaccinal anti-SARS-CoV-2 IgG titers in this group. This phenomenon was observed after primary vaccination with two doses of the ChAd vaccine and after the BNT booster.

To select the most significant variables for the final model, a multiple linear regression analysis using a backward elimination method was performed. Applied models explained 26.00% of the adjustment for results obtained 14–21 days after the 2nd dose of the ChAd vaccine and 20.00% for results achieved 21 days after the BNT booster. Since many other random variables could affect the antibodies level on the day of examination (e.g., participant’s health status on the day of blood collection, possible exposure to COVID-19 or other infections) finding an explanation of the variance in 100.00% was impossible. Also, considering our sample size, obtained adjustment ratios seem significant and worth presenting.

We found that 14–21 days after completing primary vaccination with the ChAd and 21 days after receiving the BNT booster, a significant impact on anti-SARS-CoV-2 IgG titers had non-fat parameters. In addition, backward stepwise regression analysis revealed a weak but significant correlation between NFF, i.e., body bone mass, body muscle mass, fat-free mass, and body water, and IgG antibody titers both after primary vaccination and the booster.

Similar to adipose tissue, muscle tissue is capable of secreting proteins and thus may affect the immune system’s modulation [89, 90]. Also, skeletal muscles (included in the non-fat-related factor in our study) play a significant role in this process. Decreased muscle mass may translate into a decreased immune response–which could explain our results [91, 92].

The abovementioned outcomes correspond with results from our previous research, in which we investigated a humoral response in healthcare workers primed and boosted with the BNT vaccine [38, 39]. Although our study groups consisted of males and females, they were highly feminized, which allowed us to compare the results. In our first study, we found that increased muscle mass translated positively onto the magnitude and durability of post-vaccinal humoral response, measured in anti-SARS-CoV-2 IgG antibodies. Decreased body fat mass, in turn, was positively associated with the durability of humoral response after primary vaccination: subjects with decreased BFM maintained higher anti-SARS-CoV-2 IgG titers eight months after the primary vaccination course than individuals with increased BFM [38]. However, the follow-up performed 21 days after administration of the BNT booster in this group revealed a weak positive correlation between both BFM, PMM, and anti-SARS-CoV-2 IgG titers [39].

Glowinska et al., in research on the impact of adiposity on the level of anti-SARS-CoV-2 after mRNA booster (BNT162b2 or mRNA-1273), did not find a significant correlation between those two variables [93]. It must be stressed, however, that their study group consisted of patients–males and females with end-stage kidney disease, which hinders the comparison to our group of relatively healthy individuals, and females only.

In addition, in our research, we analyzed the correlation between being naïve before the study started (understood as having anti-SARS-CoV-2 IgG titer ≥10.00 AU/mL) and the magnitude of post-vaccinal anti-SARS-CoV-2 IgG titers.

A significant impact of exposure to SARS-CoV-2 before the vaccination on vaccine-induced anti-SARS-CoV-2 IgG antibody production has been relatively well explored [94–97]. Fraley et al. investigated this matter in a feminized group of healthcare workers vaccinated with two doses of the BNT vaccine. They found that females who were convalescents before the first vaccine dose had significantly higher anti-SARS-CoV-2 IgG titers than their seronegative counterparts (i.e., naïve before the first dose). This observation was confirmed both 3 and 7 weeks after primary vaccination. Prior COVID-19 history was associated with a stronger immune response after the primary vaccination. Interestingly, in females naïve before the first dose, it was only another stimulus–i.e., second vaccine dose, that allowed them to reach similar anti-SARS-CoV-2 IgG titers as convalescents had after the first dose [98].

The results of our study confirm those findings. We found that fact of undergoing COVID-19 before the study started translated into a higher amount of anti-SARS-CoV-2 IgG titers produced after the primary vaccination course. 14–21 days after completing primary vaccination with ChAd (3^rd^ blood draw) convalescents achieved significantly higher levels of anti-SARS-CoV-2 IgG titers than non-seropositive participants.

Interestingly, in the case of the booster dose, we did not find a significant association between being naïve before the study started and the magnitude of post-booster anti-SARS-CoV-2 IgG titers. All individuals, including those who were classified as naïve before the study’s start, had developed some amounts of anti-SARS-CoV-2 IgGs during primary vaccination.

It must be added, however, that a comparison of all blood draws revealed that the median of anti-SARS-CoV-2 IgG antibody titers in our group was the highest after the BNT booster (5^th^ blood draw). It means that additional contact with SARS-CoV-2 protein in the form of the booster dose mobilized participants’ immune systems to a stronger response than after the primary vaccination. This phenomenon could be due to immune memory–the presence of memory B cells circulating in the body, capable of producing IgG in response to the stimulus, while anti-SARS-CoV-2 IgGs, circulating in the periphery, have already disappeared [99].

It is worth noting that the kinetics of adaptive immune responses to the SARS-CoV-2 vaccine is still being investigated; it requires further, long-term research to identify precisely and fully understand its mechanisms. Nevertheless, evidence-based findings in this field could translate into creating more effective strategies related to the prevention of COVID-19. Undoubtedly, data on sex-specific immune response kinetics could also enable for establishing of more optimal, personalized, and sex-tailored vaccination policy.

Another separate and crucial issue constitutes an optimal vaccination scheme. There is a mounting body of evidence indicating that the combination of vector vaccines (ChAd) with mRNA product (e.g., BNT) results in higher anti-SARS-CoV-2 IgG titers production than in case of homologous vaccination schedule [12, 100]. Due to its safety and immunogenicity, such an immunization scheme has been applied in many countries, including Poland [101, 102].

The recommendation to use a heterologous booster appears to be a beneficial strategy for stimulating and enhancing immune response [12, 103]. It becomes of even greater importance in light of recent findings about waning vaccine-induced protection over time [104–106]. It has been observed that it begins as early as one month after completing the primary vaccination; since then, it is systematically decreasing, reaching a culminating drop 6–9 months later [97, 107–109]. Gradual, but the fast-paced declining immune response after vaccination against COVID-19 has been recognized as a significant challenge for public health [110].

In addition, multiple studies report reduced immunogenicity to vaccination against COVID-19 in the elderly [111, 112]. This phenomenon seems understandable keeping in mind immunosenescence and poorer overall health conditions of older individuals which may negatively impact their adaptive immune responses. Renia et al., in their study on older vaccinees primed with the BNT, confirmed that individuals aged ≥60 years had developed lower post-vaccinal anti-SARS-CoV-2 titers and at a slower pace than their younger counterparts. It means they require more time after primary vaccination to acquire a demanded level of protection [113]. Bernal et al. reported that elderly individuals aged ≥70 years vaccinated with one dose of ChAd reached vaccine effectiveness of 60% only from 28–34 days after the vaccination [114]. Furthermore, Muller et al., in their research on the age-dependent immune response to the BNT vaccine conducted on the group of younger (<60 years) and older (>80 years) vaccinees noted that older individuals had significantly lower anti-SARS-CoV-2 IgG titers than younger subjects. However, it is worth stressing that they responded more robustly to the second vaccine dose, i.e., had a higher increase of anti-SARS-CoV-2 IgGs [115].

We did not find a correlation between advanced age (≥60 years) and anti-SARS-CoV-2 IgG concentrations on the timeline. However, this observation could result from a relatively small sample size.

Also, we did not observe a significant association between smoking and post-vaccinal response. Similar results were obtained by Kato et al., who investigated vaccine-induced anti-SARS-CoV-2 IgG titers in smokers [116]. It must be stressed, however, that most studies on that topic report significantly lower anti-SARS-CoV-2 antibody levels and more rapidly waning IgG titers in vaccinated smokers than in vaccinated non-smokers [117–120].

The magnitude and durability of the humoral immune response following vaccination also depend on inter-individual variability, i.a., genetic (HLA, major histocompatibility complex molecules class I, II), behavioral and environmental factors (such as diet, amount of vitamin C, E, D3 in the blood serum), and vaccinee’s clinical status [121–123]. Not without significance is an interval between vaccines [124].

Concluding, the public health implications of the antibody response to COVID-19 heterogeneous vaccination schemes are complex and multifaceted issue that requires further research. While the mixed vaccine schedules have shown promising results in generating a robust immune response, concerns remain regarding their safety and long-term effectiveness. Therefore, it is essential to continue monitoring post-vaccinal immunity and ensure that vaccination campaigns are implemented in a way that maximizes both their efficacy and safety.

### Study limitations

Our study has certain limitations: first of all, it has been conducted on a relatively small sample size. Also, smokers and older individuals comprised a small percentage of our study group; therefore, verifying our findings on a larger, more heterologous cohort of females would be recommended.

### Implications for public health

Our study has its strengths. To the best of our knowledge, we were among the first who used bioelectrical impedance analysis results, and not only BMI, to determine the impact of excessive fat tissue, fat- and non-fat-related parameters on anti-SARS-CoV-2 IgG titers following vaccination against COVID-19. Moreover, our study also investigated the impact of body composition parameters on post-booster humoral response. In addition, it was conducted on the population primed with vector ChAd vaccine, and findings regarding antibody response following immunization with this type of product are limited. Our research provides missing in the real world data on the magnitude and long-term durability of ChAd-induced immunity.

Undoubtedly, our paper’s strength lies in adopting a female-oriented perspective in our analyses. Our results constitute, therefore, an input to the existing gap in knowledge on sex-specific immune responses to vaccination against COVID-19.

Our findings form essential implications for public health: they highlight the evidence-based need for establishing a sex-sensitive vaccination policy, which considers sex-specific factors shaping post-vaccinal immunity. Furthermore, in the light of current demographic situation—a feminization of many social and professional groups (i.a., of older generations, professionals employed in the critical infrastructure, e.g., healthcare workers, and nations affected by the war), the implementation of female-oriented vaccination programs become particularly important.

## Conclusions

Fat- and non-fat-related components of the body as well as seropositivity before receiving the first dose of ChAd vaccine against SARS-CoV-2 have a significant impact on the level of anti-SARS-CoV-2 IgG titers in females after the primary vaccination course. Individuals with the higher amounts of non-fat-related components had on average higher anti-SARS-CoV-2 IgG titers after the booster than their counterparts with lower NFF. The fact of having reactive IgG titers before the first dose becomes irrelevant in the prediction of anti-SARS-CoV-2 IgG antibody concentrations after the BNT booster. Thus, it can be assumed that undergoing COVID-19 infection does not translate into higher levels of anti-SARS-CoV-2 IgG antibodies in boosted individuals. Body composition has a significant influence on the humoral IgG response to the booster. Its components are strictly related to biological sex. Knowledge about sex-specific differences and factors affecting immune response to SARS-CoV-2 infection and vaccination could contribute to developing more effective sex-tailored immunization programs and establishing a more optimal vaccination policy.
